# Effect of onset age on the long-term outcome of early-onset psychoses and other mental disorders: a register-based Northern Finland Birth Cohort 1986 study

**DOI:** 10.1007/s00787-023-02279-5

**Published:** 2023-08-11

**Authors:** Tuomas Majuri, Marianne Haapea, Tanja Nordström, Veera Säynäjäkangas, Kristiina Moilanen, Jonna Tolonen, Leena Ala-Mursula, Jouko Miettunen, Erika Jääskeläinen

**Affiliations:** 1 Research Unit of Population Health, Faculty of Medicine, University of Oulu, P.O.BOX 5000, 90014 Oulu, Finland; 2 Medical Research Center Oulu, Oulu University Hospital and University of Oulu, Oulu, Finland; 3 Department of Psychiatry, Oulu University Hospital, Oulu, Finland; 4 Infrastructure for Population Studies, Northern Finland Birth Cohorts, University of Oulu, Arctic Biobank, Oulu, Finland

**Keywords:** Schizophrenia, Psychosis, Early-onset, Follow-up, Outcome, Onset age

## Abstract

Psychiatric illnesses can affect the social transitions of adolescence and young adulthood, such as completing education and entering working life and relationships. However, associations between earlier onset age and long-term outcomes among those with early-onset psychoses (EOP) are unclear, as are the long-term outcomes of EOP compared to non-psychotic disorders. We used national register data of the Northern Finland Birth Cohort 1986 to detect persons with EOP and other early-onset psychiatric disorders. The long-term clinical and work-family outcomes of persons with onset age before 18 years (n = 41 psychoses, n = 495 non-psychoses) or between 18**–**22 years (n = 61 psychoses, n = 377 non-psychoses) were compared. Individuals with the onset of psychosis between 18**–**22 years had significantly more unfavourable long-term outcomes when compared to those with psychosis onset before 18 years. Persons with psychosis onset before the age of 18 years had similar outcomes to those with non-psychotic psychiatric disorder onset before 18 years regarding educational level, marital status, having children, and substance use disorders. Individuals with EOP were more often on a disability pension compared to those with other early-onset mental disorders. Adjusting for sex, educational level and substance use only slightly diluted these results. Unexpectedly, later onset age of EOP was associated with worse outcomes. Those with psychosis onset between 18**–**22 years of age are in a critical period, which underlines the importance of investing on interventions in this age group. Further studies on the effect of the onset age on later outcomes in EOP are needed.

**Supplementary Information:**

The online version contains supplementary material available at 10.1007/s00787-023-02279-5.

## Introduction

Adolescence and young adulthood are important periods when most young adults enter and complete their studies and enter working life [1]. Mental health disorders often begin already in adolescence [2] and if they emerge in this period, they can have severe effects on well-being and outcomes in later life. Adolescent mental health disorders are also risk factors for future mental distress and psychopathology [3].

In psychotic disorders, age at illness onset plays a critical role being a predictor of long-term outcomes in psychoses, with earlier age of illness onset usually associated with poorer outcomes [4–6]. The peak age at onset is 21 years for schizophrenia, schizophrenia-spectrum and other primary psychotic disorders and 19 years for acute and transient psychotic disorders [7].

The definition of early-onset psychosis (EOP) varies across studies, the most common definition being psychosis with the age of illness onset before age 18 [5, 6, 8, 9], but onset ages before 21 years [5] or even before 25 years [1] are used in some studies.

Clinical outcomes in EOP vary. Studies have reported that in cross-sectional settings 21**–**34% of individuals with EOP do not have any psychiatric treatment after 3**–**7 years of follow-up [10, 11]. Of the individuals with early-onset schizophrenia (EOS), 69% are rehospitalized after 25 years of age [1]. In EOS, 13**–**15% of individuals are in inpatient treatment, 58**–**59% are in outpatient treatment and 26**–**28% are without psychiatric care after 7**–**12 years of follow-up [12, 13].

Work-family outcomes are often unfavourable in EOP. Early-onset psychosis affects many aspects of life course including transitions to adult roles regarding family formation and entering working life after education [14]. EOS is associated with a high risk of being outside the labour market, having no secondary or higher education, and living alone [1]. Compared to all psychiatric disorders diagnosed at 10**–**20 years of age, psychoses are associated with the highest risk of long-term exclusion from education, employment or training later in adulthood [15].

Younger age of illness onset in psychoses is typically associated with poorer outcomes [4, 5] while associations between earlier onset age and outcome in EOP are inconsistent [6]. Studies on the effect of age of illness onset on very long-term outcomes in EOP and compared to other psychiatric disorders are missing. Many studies have analysed the effects of onset age on later outcomes as a continuous variable. However, associations between earlier onset age and long-term outcome among the subgroup of individuals with early-onset psychoses are unclear.

A previous study suggested studying the age of onset within the early-onset schizophrenia group to clarify possible differences in outcomes between different forms of the disorder [16]. Much is still unknown concerning detailed investigations of work-family outcomes such as education, disability pensions, and family status in EOS [16].

The study aimed to compare the long-term work-family and clinical prognosis of EOP and other early-onset psychiatric disorders with onset before 18 or between 18 and 22 years of age. The data included prospectively collected national-level register data linked to general population birth cohort with follow-up until the age of 33 years.

## Methods

### Sample

The study was based on the Northern Finland Birth Cohort 1986 (NFBC1986) [17], which is an unselected, general population sample based on 9432 live-born children with an expected date of birth between July 1st, 1985–June 30^th^, 1986, in the provinces of Oulu and Lapland [18]. The cohort members have been followed up with data collection at different ages and with individual-level linkage to registered data from various highly reliable Finnish national registers [19]. In this study, we only utilized national register data which provide up-to-date longitudinal demographic and clinical information for the cohort members.

### Case detection

Psychiatric diagnoses of NFBC1986 members were obtained from multiple national registers: the Care Register for Health Care (CRHC) [20], the Social Insurance Institution of Finland (SII) [21], the Finnish Centre for Pensions (FCP) [22], and the Register of Primary Health Care Visits [20]. See Online Resource 1 for further details.

Considering earlier literature on the definitions for early-onset psychoses [1, 5], and adolescence [23] together with the upper age limit of treatment in adolescent psychiatry services in Finland, we defined EOP as psychosis diagnosis before the age of 23 years. For comparison purposes, the same age limit was used for other psychiatric disorders in the study. To observe the effect of the age of illness onset on later outcomes in EOP, we divided both diagnostic categories into two classes including those with illness onset age before 18 years and those with illness onset between 18–22 years of age.

Cohort members diagnosed with psychosis (schizophrenia or other psychosis) or any non-psychotic psychiatric disorder before the age of 23 years based on different versions of the International Classification of Diseases (ICD-9; ICD-10) were searched from the registers. Please see Table 1 for the diagnostic categories used in the study. Psychosis diagnosis was ranked hierarchically more severe than non-psychotic disorder diagnosis. Subjects with non-psychotic disorders should not have had a diagnosis of psychosis in any of the registers, because such a diagnosis would have moved them to the psychosis group.

**Table 1:** Diagnostic categories based on ICD 9–10 used in the current study

	ICD-9 (1987**–**95)	ICD-10 (1996**–**)
Psychosis (P)	2950–2959, 2961E, 2962E, 2963E, 2964E, 2967, 297, 2988, 2989	F20, F22-F25, F28, F29, F302, F312, F315, F323, F333
Non-psychotic psychiatric disorder (NP)	295–309, 311–316 (excluding those with diagnosis of psychosis described above)	F101, F102, F111, F112, F121, F122, F131, F132, F141, F142, F151, F152, F161, F162, F171, F172, F181, F182, F191, F192, F20-F69, F80-F99 (excluding those with diagnosis of psychosis described above)

Individuals with a diagnosis of organic psychosis (e.g., ICD-10: F00-F09) or mental disability (e.g., ICD-10: F70-79) were excluded from the sample. Moreover, individuals who had deceased by the end of 2019 were excluded (information on the date of death from the Population Register) [24]. After the exclusions, we detected 102 subjects with psychosis, and 872 with a non-psychotic psychiatric disorder (NP) and these subjects formed the final sample of this study (n = 974).

In the NFBC1986, 41 persons had a psychosis diagnosis before the age of 18 years (P < 18y) and 61 persons at the age of 18–22 years (P18–22y). 495 persons had non-psychotic psychiatric disorders diagnosed before the age of 18 years (NP < 18y) and 377 persons at the age of 18–22 years (NP18–22y). Each subject in the sample was followed until the end of the follow-up (Dec 31^st^, 2019), i.e., the age of 33 years.

### Background characteristics

The following variables were used to describe the characteristics of the sample: sex, history of different psychotic and non-psychotic psychiatric disorder diagnoses, and age of illness onset. Information on these was gained from national registers (Online Resource 1). To analyse the occurrence of specific psychosis diagnoses, we used a hierarchical system, in which the main psychosis diagnosis was the disorder that had the highest position in the hierarchy based on severity (schizophrenia, schizophrenia spectrum disorder, affective psychosis, and other non-affective psychosis). The diagnoses of specific non-psychotic psychiatric disorders (depression, bipolar disorder, anxiety disorder, alcohol use disorder, cannabis use disorder, or any other substance use disorder) were analysed.

### Clinical outcomes

Data on having developed *substance use disorders* (mental and behavioural disorders due to the use of alcohol, cannabinoids, or any other substances) until 2019 were gathered from the CRHC and outpatient registers. The data on *the number of psychiatric hospital episodes* (until 2019) was obtained from the CRHC. The number of psychiatric hospital episodes due to psychosis and due to any psychiatric disorder during the latest five years of follow-up were studied.

### Work-family outcomes

The register of Statistics Finland (until 2019) was used to gain information on *educational* *level* [25]. The different educational level categories used in the study were based on the International Standard Classification of Education [26]. Basic or below level included early childhood education, primary education, and lower secondary education. Secondary level included upper secondary education and post-secondary non-tertiary education. Tertiary education included short-cycle tertiary education, Bachelor or equivalent level, Master or equivalent level, and doctoral or equivalent level.

The register of the Digital and Population Data Services Agency (until June 2016) was used to gain information on *marital status* and *having children* [24].

*Socio-economic status* at the age of 32 years (until 2018) included the following categories: farmers, entrepreneurs, upper white collar, lower white collar, manual workers, students, pensioners, and others, mostly unemployed, and was obtained from the register of Statistics Finland [27]. Socio-economic status was presented by dividing the variable into three categories: 1) white collar i.e., lower to upper white collar, 2) pensioners and 3) others i.e., farmers, entrepreneurs, manual workers, students, and others.

Information on having received *disability pension* was gathered using the registers of the Finnish Centre for Pensions (FCP) (until 2019) and Statistics Finland (socioeconomic status) until 2018. Disability pensions (permanent or fixed-term) were studied as occurring at any time point during the follow-up and at the end of the follow-up.

### Missing data

Information on socioeconomic status was missing from 2 to 5% of individuals in different diagnostic groups. Information on a disability pension at some point was missing from 0 to 3% and information on a disability pension at the end of the follow-up from 2 to 5%.

### Statistical analyses

The total numbers of psychosis and non-psychotic psychiatric disorder diagnoses from the original cohort sample before age 18 years and between ages 18–22 were calculated and presented by sex.

The background variables were presented separately for the four (P < 18y, P18–22y, NP < 18y, and NP18–22y) groups. The background characteristics of the sample were calculated using cross-tabulation (categorical variables) with the chi-square test (or Fisher’s exact test when appropriate) or median with interquartile range (continuous variables). P values < 0.05 were considered statistically significant. All tests were two-tailed.

Logistic regression was used to compare different outcome categories between the study groups. Considering earlier literature, the study groups with either higher onset age or NP diagnosis was selected as a reference category, as these groups were assumed to have most favourable outcomes. First, we conducted an unadjusted logistic regression. Then, we adjusted the regression analyses for sex (Model 1), educational level (Model 2) and any substance use disorder (Model 3) for the differences that had been statistically significant in the unadjusted analyses. The results are presented as odds ratios (ORs) with 95% confidence intervals (CIs) and p values.

In the sensitivity analyses, all statistical analyses regarding characteristics of the sample and unadjusted measures of outcome were reconducted by excluding those with psychosis or non-psychotic disorder diagnosis as early as < 13 years old from the P < 18y and NP < 18y groups and comparing the same variables between the new study groups (P13**–**18y, P18–22y, NP13**–**18y, and NP18–22y) by using logistic regression, cross-tabulation and the chi-square test (or Fisher’s exact test when appropriate) or median with interquartile range and Mann–Whitney Test.

The statistical analyses were done using IBM SPSS Statistics, version 28.

## Results

### Background characteristics

Among NFBC1986 members, 0.2% (10/4865) of males and 0.7% (31/4567) of females had a psychosis diagnosis before the age of 18 years and 0.8% of males and 0.5% of females between 18 and 22 years of age (Online Resource 2). A total of 5.1% of males and 5.4% females had NP diagnosis before the age of 18 years and 3.5% of males and 4.5% of females between 18 and 22 years.

There were fewer males (24%) among P < 18y than in other groups (45**–**62%) (Online Resource 2).

In the P < 18y group, 27% had schizophrenia or schizophrenia spectrum disorder, 27% had affective psychosis and 46% had other non-affective psychosis. In the P18–22y group, 15% had schizophrenia or schizophrenia spectrum disorder, 26% had affective psychosis and 59% had other non-affective psychosis. Most of the persons in NP groups had registered diagnosis of depression (28**–**45%) or anxiety disorder (18**–**50%) (Online Resource 2).

The median age of psychosis onset was 16 years in P < 18y and 21 years in P18–22y. The median age of illness onset was 14 years in NP < 18y and 20 years in NP18–22y.

Until the end of the follow-up, only a few psychosis diagnoses had changed to more severe diagnoses in the hierarchy (Online Resource 2). Conversion of the psychosis diagnoses is presented in Online Resource 3.

### Clinical outcomes during the follow-up

Among those with P < 18y, 10% and among P18–22y, 23% had psychiatric hospital episodes due to psychosis during the latest five years of follow-up (Table 2). In P < 18y, 15% and among P18–22y, 33% had hospital episodes due to any psychiatric disorder during the latest five years of follow-up. Only the difference in hospitalizations due to any psychiatric disorder was statistically significant between psychosis groups in crude analyses and after adjusting for educational level and substance use disorder, the statistical significance was lost (Online Resource 4).

**Table 2:** Clinical outcomes during the follow-up, frequencies, percentages, p-values and unadjusted odds ratios in relation to the reference groups

Variable	Psychosis < 18 years (n = 41)	Psychosis 18–22 years (n = 61)	Non-psychotic psychiatric disorder < 18 years (n = 495)	Non-psychotic psychiatric disorder 18–22 years (n = 377)	P < 18y vs P18–22y^1^			P < 18y vs. NP < 18y^1^			P18–22y vs. NP18–22y^1^			NP < 18y vs. NP18–22y^1^		
					Crude OR (95% CI)	p-value		Crude OR (95% CI)	p-value		Crude OR (95% CI)	p-value		Crude OR (95% CI)	p-value	
Psychiatric hospital episodes, psychosis, n (%)^a^	4 (9.8)	14 (23.0)	13 (2.6)	12 (3.2)	0.36 (0.11–1.20)	0.100		4.01 (1.25–12.91)	0.020		9.06 (3.96–20.75)	< 0.001		0.82 (0.37–1.82)	0.626	
Psychiatric hospital episodes, any psychiatric, n (%)^a^	6 (14.6)	20 (32.8)	35 (7.1)	46 (12.2)	0.35 (0.13–0.97)	0.044		2.25 (0.89–5.72)	0.088		3.51 (1.89–6.51)	< 0.001		0.55 (0.35–0.87)	0.011	
Substance use disorders during the follow-up, n (%)																
Any substance use disorder, n (%)	7 (17.1)	23 (37.7)	103 (20.8)	89 (23.6)	0.34 (0.13–0.89)	0.028		0.78 (0.34–1.82)	0.570		1.96 (1.11–3.46)	0.021		0.85 (0.62–1.17)	0.323	
Alcohol use disorder, n (%)	5 (12.2)	19 (31.1)	87 (17.6)	65 (17.2)	0.31 (0.10–0.91)	0.032		0.65 (0.25–1.71)	0.383		2.17 (1.19–3.97)	0.012		1.02 (0.72–1.46)	0.897	
Cannabis use disorder, n (%)	2 (4.9)	3 (4.9)	7 (1.4)	12 (3.2)	0.99 (0.16–6.21)	0.993		3.58 (0.72–17.80)	0.120		1.57 (0.43–5.75)	0.493		0.44 (0.17–1.12)	0.084	
Other substance use disorder, n (%)	5 (12.2)	10 (16.4)	40 (8.1)	46 (12.2)	0.71 (0.22–2.25)	0.558		1.58 (0.59–4.25)	0.365		1.41 (0.67–2.97)	0.365		0.63 (0.41–0.99)	0.045	

*OR* odds ratio, *CI* confidence interval

^1^Reference category

^a^Psychiatric hospital episodes counted only for the last five years of follow-up (2015–2019)

Persons with NP < 18y had less psychiatric hospitalizations compared to NP18-22y (OR: 0.55; 95% CI: 0.35–0.87). Among persons with NP < 18y, 7% and in NP18–22y, 12% had psychiatric hospital episodes due to any psychiatric disorder during the latest five years of follow-up.

The proportion of alcohol use disorders was statistically significantly higher (31%) in the P18–22y group compared to other groups (12**–**18%). The proportions of disorders due to the use of cannabinoids were 1**–**5%. The rates of disorders due to the use of any other substances were 8**–**16%. Those with NP < 18y had statistically significantly less use of other substances than the NP18–22y group. After adjustment for sex, the statistical significances in substance use disorders and alcohol use disorders between the P < 18y and P18-22y and in substance use disorders between the P18-22y and NP18-22y disappeared. After adjusting for educational level, the statistical significances in alcohol use disorders between the P < 18y and P18-22y and in any substance use disorders and alcohol use disorders between the P18-22y and NP18-22y disappeared (Online Resource 4).

### Work-family outcomes

Secondary education was the most common educational level in all groups (51**–**58%) (Table 3). Tertiary education was attained by 13% of individuals with P18–22y and 23**–**28% of individuals in other groups. Only the difference in tertiary education between P18–22y and NP18–22y was statistically significant (Table 3).

**Table 3:** Work-family outcomes during the follow-up, frequencies, percentages, p-values and unadjusted odds ratios in relation to the reference groups

Variable	Psychosis < 18 years (n = 41)	Psychosis 18–22 years (n = 61)	Non-psychotic psychiatric disorder < 18 years (n = 495)	Non-psychotic psychiatric disorder 18–22 years (n = 377)	P < 18y vs. P18–22y^1^		P < 18y vs. NP < 18y^1^		P18–22y vs. NP18–22y^1^		NP < 18y vs. NP18–22y^1^	
					Crude OR (95% CI)	p-value	Crude OR (95% CI)	p-value	Crude OR (95% CI)	p-value	Crude OR (95% CI)	p-value
Educational level, n (%)^a^												
Basic or below	10 (24.4)	18 (29.5)	95 (19.2)	60 (15.9)	1		1		1		1	
Secondary	21 (51.2)	35 (57.4)	286 (57.8)	213 (56.5)	1.08 (0.42–2.78)	0.873	0.70 (0.32–1.53)	0.370	0.55 (0.29–1.04)	0.064	0.85 (0.59–1.23)	0.381
Tertiary	10 (24.4)	8 (13.1)	114 (23.0)	104 (27.6)	2.23 (0.67–7.54)	0.189	0.83 (0.33–2.08)	0.697	0.26 (0.11–0.63)	0.003	0.69 (0.46–1.05)	0.085
Marital status, n (%)^b^						0.001		0.226		0.001		0.479
Single/divorced/separated/widowed	27 (65.9)	58 (95.1)	369 (74.5)	273 (72.4)	1		1		1		1	
Married/registered	14 (34.1)	3 (4.9)	126 (25.5)	104 (27.6)	10.03 (2.66–37.83)		1.52 (0.77–2.99)		0.14 (0.04–0.44)		0.90 (0.66–1.21)	
Having children, n (%)^b^	17 (41.5)	13 (21.3)	228 (46.1)	181 (48.0)	2.62 (1.09–6.26)	0.031	0.83 (0.44–1.58)	0.571	0.29 (0.15–0.56)	< 0.001	0.93 (0.71–1.21)	0.568
Socio-economic status, n (%)^c^												
White collar	12 (30.0)	10 (16.9)	156 (32.7)	138 (38.3)	1		1		1		1	
Farmer/entrepreneur/manual worker/student/other	17 (42.5)	30 (50.8)	285 (59.7)	201 (55.8)	0.47 (0.17–1.32)	0.153	0.78 (0.36–1.67)	0.514	2.06 (0.98–4.35)	0.058	1.25 (0.94–1.68)	0.128
Pensioner	11 (27.5)	19 (32.2)	36 (7.5)	21 (5.8)	0.48 (0.16–1.48)	0.202	3.97 (1.62–9.72)	0.003	12.49 (5.11–30.49)	< 0.001	1.52 (0.85–2.72)	0.163
Disability pension at some point, n (%)	15 (36.6)	27 (45.8)	55 (11.5)	53 (14.4)	0.68 (0.30–1.55)	0.361	4.46 (2.23–8.93)	< 0.001	5.00 (2.77–9.01)	< 0.001	0.77 (0.51–1.15)	0.198
Disability pension at the end of the follow-up, n (%)	11 (27.5)	20 (33.9)	40 (8.4)	26 (7.2)	0.74 (0.31–1.78)	0.501	4.14 (1.93–8.91)	< 0.001	6.59 (3.37–12.88)	< 0.001	1.18 (0.70–1.97)	0.537

*OR* odds ratio, *CI* confidence interval

^1^Reference category

^a^At 2019, ^b^At June 2016, ^c^At 2018

Marital status was married or registered cohabitation for 5% of those with P18–22y and 26**–**34% for individuals in other groups. Persons with P < 18y had tenfold odds of getting married during the follow-up compared to those with older onset age of psychosis. Those with P18-22y had 86% lower odds for marriage than those with NP18-22y. In terms of offspring, persons in the P18–22y group had significantly less often children (21%) than individuals in other groups (41**–**48%). After adjusting for sex, the statistical significance in having children between the P < 18y and P18-22y disappeared (Online Resource 5). In all groups, females had more often children (30–58%) than males (16–36%).

The persons in all groups were most often (43–60%) farmers, entrepreneurs, manual workers, students, or others.

Among persons with psychoses, 37**–**46% had been on a disability pension at some point whereas the corresponding rate for the NP groups was 12**–**14%. At the end of the follow-up, the proportions of disability pensions were significantly higher for psychosis groups (28**–**34%) than for non-psychosis groups (7**–**8%). Compared to corresponding NP groups, those with P < 18y had fourfold odds and those with P18-22y had fivefold odds of being on a disability pension at some point. The odds ratios for disability pension at the end of the follow-up in these groups were 4.1 and 6.6, respectively. Onset age did not have an effect on the rate of disability pensions within either psychoses or non-psychoses.

### Sensitivity analysis

When excluding from the analyses those with childhood psychosis or non-psychosis (i.e., diagnosis before age 13 years), the new sample sizes were n = 37 for P13**–**18y and n = 294 for NP13**–**18y, and n = 769 for the total sample. The results of the sensitivity analysis were mainly similar to the main analyses. The differences in the number of the psychiatric hospital episodes due to any psychiatric disorder between the NP groups and in disorders due to the use of any other substances between NP groups lost statistical significance in the sensitivity analyses (Online Resources 6–9).

## Discussion

### Main findings

To our knowledge, this was the first population-level follow-up study to assess in detail the effect of onset age on long-term outcomes of early-onset psychoses and other psychiatric disorders by comparing outcomes by onset ages before 18 and from 18 to 22 years, potentially affecting important social transitions. The study presents a novel finding showing that psychotic disorder before age of 18 years does not unambiguously have a poorer prognosis when compared to psychosis with an onset age between 18 and 22 years of age. Instead, individuals with earlier onset of psychosis (before 18 years) had better long-term outcomes in terms of work-family outcomes. In terms of clinical outcomes, those with psychosis onset age between 18 and 22 years had more often developed alcohol use disorders compared to those with onset age before 18 years, whereas the number of hospitalizations did not significantly vary by onset age. Regarding educational level, marital status, having children, and developing substance use disorders, persons with psychosis diagnosis before the age of 18 years had similar outcomes to those with non-psychotic psychiatric disorder onset before the age of 18 years. Individuals with EOP had more disability pensions compared to other early-onset mental disorders. Compared to persons with psychosis onset age before 18 years of age, those with psychosis onset between 18 and 22 years of age were more often males, had more alcohol use disorders and lower educational level. Sex, educational level and substance use disorders were found to moderate some of the other outcomes between psychosis groups with different onset ages.

### Comparison to previous studies

We found a total rate of any psychosis before age 23 years being 1.0% for males and 1.2% for females. Corresponding rates of non-psychotic disorders before age 23 years were 8.6% for males and 9.9% for females. Our figures are quite well in line with the Finnish 1981 Birth Cohort study reporting that 1.5% of males and 0.8% of females are in psychiatric hospital treatment due to psychosis and 6.2% of males and 4.1% of females due to any psychiatric disorder between ages 13 and 24 [28]. Compared to the Finnish Birth Cohort study [28], the higher rates of non-psychotic disorders could be explained by using more numerous national registers in the case detection phase of our study, not only information on hospitalizations..

In both psychosis and non-psychotic disorders, those with older age of illness onset had more psychiatric hospital episodes due to their respective disorders during the latest five years of the follow-up. Adjusted analysis showed that in psychoses this may be moderated by the effect of educational level and substance use disorders. However, the difference was statistically significant in NP groups only. The number of psychiatric inpatient services in Finland has been decreasing for several decades [29] and due to period effect, outcomes related to hospitalizations has to be interpreted carefully. Younger age of illness onset has previously been associated with more hospitalizations in schizophrenia [4]. Moreover, an Israeli study has reported a linear trend between onset age and hospitalizations showing increased hospital use for individuals with earlier onset [30]. A recent study found that compared to adult onset, those with EOS have more inpatient days in the first two years after diagnosis, but long-term outcome in terms of duration and annual rates of inpatient treatment between early-onset (< 18 years of age) and adult-onset disorders did not differ thereafter [16]. A recent meta-analysis found that 55% of individuals with first-episode psychosis were hospitalized at least once during an average follow-up length of seven years [31]. Adherence to psychiatric inpatient and outpatient treatment has been found associating with better educational and occupational outcomes in early-onset schizophrenia [1].

Due to focusing on the latest five years of follow-up, our numbers of psychotic individuals without psychiatric hospital episodes due to psychosis (77–90%) somewhat differ from the results of previous studies reporting that 21**–**34% of individuals with EOP are not in any psychiatric care after an average follow-up time of 3**–**12 years across studies [10–13] and a recent study reporting that 69% of those with EOS are rehospitalized after their 25^th^ birthday [1]. Individuals with EOS who do not need psychiatric inpatient or outpatient treatment after 25 years of age most probably have milder diseases and naturally have better occupational, educational, and social outcomes compared to those with a more chronic course of illness [1].

The cumulative prevalences of substance use disorders in our study were higher in psychoses than in NP. This finding is in line with earlier literature reporting higher rates of any co-occurring substance use disorder in psychotic disorders than in other mental disorders [32]. However, after adjusting for sex and educational level, most of the differences related to substance use disappeared emphasizing the effect of these factors. The higher number of substance use disorders, mainly due to use of alcohol, among those with later onset age of psychosis may be associated with the worse later outcomes in this age group found in this study. Substance use disorders are a growing problem among individuals with psychiatric disorders, particularly among those with psychoses [32].

Adjusted analysis showed that higher number of females in the P < 18y category (76%) compared to the P18–22y category (38%) may partly explain the differences in outcomes between psychosis groups. Female sex has typically been linked with better outcomes in schizophrenia at the outset of the illness [33] and with better outcomes in EOS in some studies [5].

In terms of achieving educational level, persons with psychosis onset at 18–22 years of age presented with the highest rate of only basic or below education (30%) and the lowest rate of tertiary education (13%) completed. In other study groups, 16**–**24% of individuals had completed only basic level, and 23**–**28% tertiary level. These results are in line with previous studies showing poorer educational outcomes for those with EOP compared to other early-onset psychiatric disorders [15, 34]. However, in our study, those with psychosis onset between 18 and 22 years of age showed poorer educational outcomes than those with psychosis diagnosis before 18 years, contradicting previous studies of EOP [6] and studies comparing EOP and adult-onset psychoses (AOP) [16]. This may be partly explained by our study focusing on the cut-off between traditional definitions for EOP and AOP instead of comparing these two forms of the disorder with their most commonly used definitions.

Persons with psychosis onset at 18–22 years included significantly more individuals who were not in a relationship (95%) compared to rest of the sample (66**–**75%). The outcomes related to the marital status did not change after different adjustments. A Chinese study reported that 21% of individuals with EOS (< 18 years old) had never been married [35] whereas another study found that among persons with EOP, 11% were married and 36% in a romantic relationship [36]. Our results on the effect of onset age on later marital status in psychoses differ somewhat from previous studies linking later onset ages to being more often married [37] and better social outcomes [4] in schizophrenia. Some studies have linked later onset age to better social functioning also in EOP [6].

The definition of EOP and EOS varies across studies. Our study included only individuals with psychosis onset before 23 years, which affects the comparability of the current study with studies also comprising individuals with adult-onset psychoses or studies including only those with onset before age 18 years as well as studies including only schizophrenia patients. The potential differences in durations of untreated psychosis may also have influenced on the formation of the study groups by prolonging the start of the treatment and thus registered diagnosis for those with psychosis onset at 18–22 years associating with worse later outcomes.

In terms of offspring, those with P18–22y significantly more often did not have children (79%) compared to other groups (52**–**59%). Previous studies have shown an association between earlier onset of psychosis and reduced fecundity [38]. In schizophrenia, men typically have reduced fertility compared to women [39]. In our study, the unbalanced number of women (76% of those with P < 18y and 38% of those with P18–22y) between psychosis groups affected the findings regarding having children, as seen in the adjusted analysis.

Disability pensions during the follow-up were more common among persons with psychoses (37**–**46%) compared to NP categories (12**–**14%). These results are in line with the previous studies reporting EOS being associated with a higher risk of being outside the labour market [1] and being unemployed [15, 34] compared to other psychiatric disorders. A review of the predictors of different outcomes in EOP reported better occupational functioning to be predicted by older age at onset [6].

### Strengths and limitations

A general population sample from the NFBC1986 with over 30 years of lifetime follow-up offers a comprehensive view of the long-term outcomes of EOP as compared to non-psychotic psychiatric disorders. A review of EOP suggested studying outcomes in national registers in order to avoid potential sample bias caused by hospital recruitment [5]. As suggested, the use of prospectively collected and extensive register data on different work-family and clinical outcomes is another strength of the study. Studying outcomes within and between onset age-based categories of psychosis and NP groups enabled comparing the courses of these disorders.

False-positive EOP diagnoses due to diagnostic practices and registration errors in outpatient settings have been reported [40]. Using data from multiple national registers in the case detection phase, we were able to minimize the number of potential misdiagnoses. Moreover, using sensitivity analysis excluding the very early onset psychoses and non-psychotic psychiatric disorders, we were able to exclude some potential childhood misdiagnoses and make the comparison between psychosis and NP groups more suitable.

The study has some limitations. The unbalanced number of males and females between the psychosis groups influenced the findings, emphasizing poorer outcomes for the P18–22y group with a greater number of males. However, by adjusted analyses, we were able to consider the effect of sex, educational level and substance use on the results. Furthermore, the study excluded psychoses due to substance use, which may be common in adolescence. This preference was due to an intention to focus on non-organic psychoses and facilitate comparison with previous studies.

Another limitation is the small sample size of individuals in the psychosis groups. Due to the small sample size, we were neither able to study outcomes between subclasses such as schizophrenia and the other inherently heterogeneous psychoses nor to study predictors of outcomes. Non-schizophrenia diagnoses have been reported to be associated with better outcomes of EOP in some studies [5, 6]. However, we wanted to study individuals with psychotic disorders instead of concentrating too much on specific diagnoses. That way, we also aimed to provide valuable general information on the outcomes of psychotic disorders for clinicians, patients and family members. Moreover, register data offer only general viewpoints on the outcomes of psychiatric disorders and do not provide a more comprehensive picture, which could have been collected with questionnaires if the challenges of generally poor response rates among psychiatric patients could be overcome.

### Clinical implications

The age of onset indeed seems to play a significant role influencing later outcomes of psychosis and would merit more active consideration when planning treatment and rehabilitation. Among those with EOP, typical adolescence-related developmental tasks such as the act of becoming independent, development of personality, and attaining age-dependent goals may be disturbed by the illness and its consequences [2]. In addition, brain development is still ongoing [41]. Those with later onset ages at adulthood may have already transitioned to adult roles including family formation and entering working life. Studying age as a categorical variable revealed age-specific differences in the course of psychosis. Many previous studies have compared EOP to adult-onset psychosis whereas we focused on comparing long-term outcomes of EOP in two age categories of adolescence due to a lack of pre-existing literature. Earlier studies have mainly drawn the line between EOP and AOP at 18 years based on the traditional definition of legal age whereas we wanted to study this cut-off between EOP and AOP by stretching the upper age limit of EOP to 23 years so as to also take into consideration brain development after 18 years of age. This choice was made also to align with the age boundaries of the Finnish treatment practices of adolescent psychiatric patients.

One possible explanation for differences in outcomes between psychosis groups may originate from society. Based on legislation and health care practices, underaged persons are covered by school health care, which may help succeed in screening individuals at risk of psychosis and accelerate the provision of the interventions and treatments needed. Those with psychosis onset at 18–22 years are in an important transition phase in which they may no longer be covered by the school health care system, and since they are not yet in working life, they are outside the occupational health care system. This may lead to a longer duration of untreated psychosis and thus, to worse later outcomes for those with onset age between 18 and 22 years. A longer duration of untreated psychosis has been linked with poorer long-term outcomes in schizophrenia, emphasizing the importance of interventions for shortening these periods [42]. Longer duration of untreated psychosis has been found to predict worse functional, clinical and cognitive outcomes also in early-onset psychosis [6]. Early intervention services have been found to be superior to treatment as usual in early-phase psychosis [43]. The poor work-family and clinical outcomes of EOP found in the current study emphasize the need for early interventions to prevent young adults from being waylaid from reaching the social translational milestones that are typically attained in young adulthood. The results indicate a specific need for interventions in outpatient settings for young adults at risk of psychosis in the important transformation phase between 18 and 22 years of age. Moreover, those who already have been diagnosed with psychoses and receiving treatment, can be successfully helped to gain and retain employment for example with Individual Placement and Support practices [44]. Due to a high number of substance use disorders among those with psychosis onset between 18 and 22 years of age, new integrated approaches combining psychiatric and addiction services for this age group are needed to offer adequate treatments for individuals with dual pathology.

## Conclusion

The outcomes of EOP are not similar for everyone. Illness onset before the age of 18 years does not necessarily associate with worse outcomes. The time between 18 and 22 years of age is an important transformation phase that may be disturbed by psychosis onset leading to poor long-term outcomes. Further studies in different age groups are needed on the prognosis and predictors of EOP to clarify onset age-related differences in the course and possible different forms of the disorder.

### Supplementary Information

Below is the link to the electronic supplementary material.

Supplementary file1 (PDF 92 KB)

Supplementary file2 (PDF 132 KB)

Supplementary file3 (PDF 51 KB)

Supplementary file4 (PDF 64 KB)

Supplementary file5 (PDF 74 KB)

Supplementary file6 (PDF 133 KB)

Supplementary file7 (PDF 52 KB)

Supplementary file8 (PDF 65 KB)

Supplementary file9 (PDF 77 KB)

## Declarations

### Data availability

NFBC data is available from the University of Oulu, Infrastructure for Population Studies. Permission to use the data can be applied for research purposes via electronic material request portal. In the use of data, we follow the EU general data protection regulation (679/2016) and Finnish Data Protection Act. The use of personal data is based on cohort participants written informed consent at their latest follow-up study, which may cause limitations to its use. Please, contact the NFBC project center (NFBCprojectcenter@oulu.fi) and visit the cohort website (www.oulu.fi/nfbc) for more information.
