# Supplementary material for: Effect of onset age on the long-term outcome of early-onset psychoses and other mental disorders: a register-based Northern Finland Birth Cohort 1986 study
Source: Eur Child Adolesc Psychiatry. 2023 Aug 11;33(6):1741–53. doi: 10.1007/s00787-023-02279-5 (PMC11211101; doi:10.1007/s00787-023-02279-5)
Supplement: Supplementary file 1 — Supplementary file1 (PDF 92 KB) [file 787_2023_2279_MOESM1_ESM.pdf]

European Child & Adolescent Psychiatry

Effect of onset age on the long-term outcome of early-onset psychoses and other mental disorders: a register based Northern Finland Birth Cohort 1986 study

Tuomas Majuri[1] · Marianne Haapea · Tanja Nordström · Veera Säynäjäkangas · Kristiina Moilanen · Jonna Tolonen · Leena Ala-Mursula · Jouko Miettunen · Erika Jääskeläinen

[1]Research Unit of Population Health, University of Oulu, Oulu, Finland.

Corresponding author:
M.D. Tuomas Majuri,
email tuomas.majuri@student.oulu.fi

Online supplement 1

Methods

Case detection

Psychiatric diagnoses of NFBC1986 members were obtained from multiple national registers. The Care Register for Health Care (CRHC) was used to find diagnoses of all general and psychiatric hospitalizations (data used for this study 1994–2019) and visits to specialized outpatient care in 1998–2019 [1]. The data on diagnoses was complemented using register information of the Social Insurance Institution of Finland (SII) [2] on received special drug reimbursement (2001–2005), and information from the Finnish Centre for Pensions (FCP) [3] on diagnoses for receiving disability pensions (1994–2019). The Register of Primary Health Care Visits (2011–2019) was also used to find outpatient diagnoses in primary care [1].

Background characteristics

Sex was based on national population register information.

History of different psychotic and non-psychotic psychiatric disorder diagnoses and *age of illness onset* were obtained by using the CRHC, the register of the FCP and the SII registers of reimbursable medicines, and Finnish outpatient registers. Age of illness onset was defined as the age of the first occurrence of psychosis or non-psychotic psychiatric disorders in the registers. *Psychosis diagnoses.* To analyse the occurrence of specific psychosis diagnoses, we used a hierarchical system, in which the main psychosis diagnosis was the disorder that had the highest position in the hierarchy based on severity. The hierarchical order of diagnoses was from the most severe disorder to the least severe disorder in the following order: schizophrenia, schizophrenia spectrum disorder, affective psychosis, and other non-affective psychosis. *Non-psychotic psychiatric diagnoses.* The diagnoses of specific non-psychotic psychiatric disorders (depression, bipolar disorder, anxiety disorder, alcohol use disorder, cannabis use disorder, or any other substance use disorder) were gathered from the national registers.

1. Finnish Institute for Health and Welfare (2021) Register descriptions. https://thl.fi/en/web/thlfi-en/statistics-and-data/data-and-services/register-descriptions. Accessed 17 Sept 2021
2. The Social Insurance Institution of Finland (2021) Statistics. https://www.kela.fi/web/en/statistics. Accessed 17 Sept 2021
3. Finnish Centre for Pensions (2021) Statistics. https://www.etk.fi/en/research-statistics-and-projections/statistics/. Accessed 17 Sept 2021
