# Supplementary material for: Effect of onset age on the long-term outcome of early-onset psychoses and other mental disorders: a register-based Northern Finland Birth Cohort 1986 study
Source: Eur Child Adolesc Psychiatry. 2023 Aug 11;33(6):1741–53. doi: 10.1007/s00787-023-02279-5 (PMC11211101; doi:10.1007/s00787-023-02279-5)
Supplement: Supplementary file 2 — Supplementary file2 (PDF 132 KB) [file 787_2023_2279_MOESM2_ESM.pdf]

European Child & Adolescent Psychiatry

Effect of onset age on the long-term outcome of early-onset psychoses and other mental disorders: a register based Northern Finland Birth Cohort 1986 study

Tuomas Majuri[1] · Marianne Haapea · Tanja Nordström · Veera Säynäjäkangas · Kristiina Moilanen · Jonna Tolonen · Leena Ala-Mursula · Jouko Miettunen · Erika Jääskeläinen

[1]Research Unit of Population Health, University of Oulu, Oulu, Finland.

Corresponding author:
M.D. Tuomas Majuri,
email tuomas.majuri@student.oulu.fi

Online supplement 2

Online supplement table 1. Characteristics of the sample

Variable	Psychosis <18 years (n=41)	Psychosis 18–22 years (n=61)	Non-psychotic psychiatric disorder <18 years (n=495)	Non-psychotic psychiatric disorder 18–22 years (n=377)	P<18y vs. P18–22y, p-value	P<18y vs. NP<18y, p-value	P18–22y vs. NP18–22y, p-value	NP<18y vs. NP18–22y, p-value
**Sex, n (%)**					<0.001	0.001	0.014	0.148
Male	10 (24.4)	38 (62.3)	249 (50.3)	171 (45.4)				
Female	31 (75.6)	23 (37.7)	246 (49.7)	206 (54.6)				
**Hierarchical psychosis diagnosis, n (%)**[a]					0.238[b]			
Schizophrenia	7 (17.1)	8 (13.1)						
Schizophrenia spectrum disorder	4 (9.8)	1 (1.6)						
Affective psychosis	11 (26.8)	16 (26.2)						
Other non-affective psychosis	19 (46.3)	36 (59.0)						
**Non-psychotic psychiatric disorder diagnosis, n (%)**[a]								
Depression	18 (43.9)	24 (39.3)	137 (27.7)	171 (45.3)				
Bipolar disorder	4 (9.8)	4 (6.6)	3 (0.6)	11 (2.9)				
Anxiety disorder	11 (26.8)	20 (32.8)	89 (18.0)	188 (49.9)				
Alcohol use disorder	3 (7.3)	13 (21.3)	42 (8.5)	48 (12.7)				
Cannabis use disorder	0 (0.0)	1 (1.6)	2 (0.4)	6 (1.6)				
Other substance use disorder	1 (2.4)	7 (11.5)	8 (1.6)	22 (5.8)				
**Age of illness onset, psychosis, Md (IQR)**	15.9 (14.7-17.0)	20.6 (19.6-21.4)						
**Age of illness onset, non-psychotic psychiatric disorder, Md (IQR)**	15.1 (13.7-16.0)	19.8 (18.1-21.0)	14.2 (9.0-16.0)	20.3 (19.3-21.6)				
**Psychosis diagnosis at the end of the follow-up, n (%)**					0.448[b]	0.312[b]	0.249[b]	0.167[b]
Schizophrenia	9 (22.0)	19 (31.1)	6 (24.0)	6 (21.4)				
Schizophrenia spectrum disorder	5 (12.2)	3 (4.9)	4 (16.0)	4 (14.3)				

Affective psychosis	11 (26.8)	13 (21.3)	2 (8.0)	9 (32.1)
Other non-affective psychosis	16 (39.0)	26 (42.6)	13 (52.0)	9 (32.1)

[a]During the time leading for belonging to the respective study group, [b]P-value presented by Fisher's exact test

Md median, *IQR* interquartile range
