# Supplementary material for: Effect of onset age on the long-term outcome of early-onset psychoses and other mental disorders: a register-based Northern Finland Birth Cohort 1986 study
Source: Eur Child Adolesc Psychiatry. 2023 Aug 11;33(6):1741–53. doi: 10.1007/s00787-023-02279-5 (PMC11211101; doi:10.1007/s00787-023-02279-5)
Supplement: Supplementary file 4 — Supplementary file4 (PDF 64 KB) [file 787_2023_2279_MOESM4_ESM.pdf]

European Child & Adolescent Psychiatry

Effect of onset age on the long-term outcome of early-onset psychoses and other mental disorders: a register based Northern Finland Birth Cohort 1986 study

Tuomas Majuri[1] · Marianne Haapea · Tanja Nordström · Veera Säynäjäkangas · Kristiina Moilanen · Jonna Tolonen · Leena Ala-Mursula · Jouko Miettunen · Erika Jääskeläinen

[1]Research Unit of Population Health, University of Oulu, Oulu, Finland.

Corresponding author:
M.D. Tuomas Majuri,
email tuomas.majuri@student.oulu.fi

Online supplement 4

Online supplement table 3. Clinical outcomes during the follow-up, adjusted odds ratios in relation to the reference groups (=1)

Variable	P<18y vs. P18–22y[1]			P<18y vs. NP<18y[1]			P18–22y vs. NP18–22y[1]			NP<18y vs. NP18–22y[1]		
	**Adjusted OR (95% CI)**											
	**Model 1[a]**	**Model 2[b]**	**Model 3[c]**	**Model 1[a]**	**Model 2[b]**	**Model 3[c]**	**Model 1[a]**	**Model 2[b]**	**Model 3[c]**	**Model 1[a]**	**Model 2[b]**	**Model 3[c]**
**Psychiatric hospital episodes, psychosis[d]**	-	-	-	5.26 (1.55-17.91)	3.92 (1.17-13.15)	4.80 (1.41-16.33)	8.23 (3.56-19.0)	8.31 (3.58-19.29)	8.61 (3.73-19.88)	-	-	-
**Psychiatric hospital episodes, any psychiatric[d]**	0.32 (0.11-0.96)	0.39 (0.14-1.10)	0.43 (0.15-1.24)	-	-	-	3.29 (1.77-6.15)	2.99 (1.59-5.63)	3.09 (1.59-5.99)	0.53 (0.33-0.84)	0.50 (0.31-0.80)	0.55 (0.34-0.90)
**Substance use disorders during the follow-up**												
Any substance use disorder	0.43 (0.16-1.20)	0.36 (0.13-0.97)	-	-	-	-	1.77 (0.99-3.15)	1.53 (0.84-2.78)	-	-	-	-
Alcohol use disorder	0.34 (0.11-1.05)	0.32 (0.11-1.00)	-	-	-	-	1.93 (1.04-3.57)	1.74 (0.93-3.26)	-	-	-	-
Cannabis use disorder	-	-	-	-	-	-	-	-	-	-	-	-
Other substance use disorder	-	-	-	-	-	-	-	-	-	0.61 (0.39-0.96)	0.55 (0.35-0.88)	-

[1]Reference category

[a]Model 1: adjusted for sex, [b]Model 2: adjusted for educational level, [c]Model 3: adjusted for any substance use disorder, [d]Psychiatric hospital episodes counted only for the last five years of follow-up (2015-2019)

OR odds ratio, *CI* confidence interval
