# Supplementary material for: Effect of onset age on the long-term outcome of early-onset psychoses and other mental disorders: a register-based Northern Finland Birth Cohort 1986 study
Source: Eur Child Adolesc Psychiatry. 2023 Aug 11;33(6):1741–53. doi: 10.1007/s00787-023-02279-5 (PMC11211101; doi:10.1007/s00787-023-02279-5)
Supplement: Supplementary file 5 — Supplementary file5 (PDF 74 KB) [file 787_2023_2279_MOESM5_ESM.pdf]

European Child & Adolescent Psychiatry

Effect of onset age on the long-term outcome of early-onset psychoses and other mental disorders: a register based Northern Finland Birth Cohort 1986 study

Tuomas Majuri[1] · Marianne Haapea · Tanja Nordström · Veera Säynäjäkangas · Kristiina Moilanen · Jonna Tolonen · Leena Ala-Mursula · Jouko Miettunen · Erika Jääskeläinen

[1]Research Unit of Population Health, University of Oulu, Oulu, Finland.

Corresponding author:
M.D. Tuomas Majuri,
email tuomas.majuri@student.oulu.fi

Online supplement 5

Online supplement table 4. Work-family outcomes during the follow-up, adjusted odds ratios in relation to the reference groups (=1)

Variable	**P<18y vs. P18–22y[1]**			**P<18y vs. NP<18y[1]**			**P18–22y vs. NP18–22y[1]**			**NP<18y vs. NP18–22y[1]**		
	**Adjusted OR (95% CI)**											
	**Model 1[a]**	**Model 2[b]**	**Model 3[c]**	**Model 1[a]**	**Model 2[b]**	**Model 3[c]**	**Model 1[a]**	**Model 2[b]**	**Model 3[c]**	**Model 1[a]**	**Model 2[b]**	**Model 3[c]**
**Educational level[d]**												
Basic or below	1	1	1	1	1	1	1	1	1	1	1	1
Secondary	-	-	-	-	-	-	-	-	-	-	-	-
Tertiary	-	-	-	-	-	-	0.30 (0.12-0.76)	-	0.31 (0.12-0.78)	-	-	-
**Marital status[e]**												
Single/divorced/separated/widowed	1	1	1	1	1	1	1	1	1	1	1	1
Married/registered	11.31 (2.70-47.48)	10.60 (2.77-40.63)	9.53 (2.47-36.75)	-	-	-	0.15 (0.05-0.49)	0.15 (0.05-0.51)	0.15 (0.05-0.48)	-	-	-
**Having children[e]**	1.86 (0.73-4.75)	2.72 (1.12-6.60)	2.64 (1.08-6.49)	-	-	-	0.33 (0.17-0.63)	0.30 (0.16-0.57)	0.30 (0.15-0.56)	-	-	-
**Socio-economic status[f]**												
White collar	1	1	1	1	1	1	1	1	1	1	1	1
Farmer/entrepreneur/manual worker/student/other	-	-	-	-	-	-	-	-	-	-	-	-
Pensioner	-	-	-	5.32 (2.10-13.52)	3.81 (1.41-10.27)	4.13 (1.67-10.20)	10.85 (4.34-27.15)	11.31 (4.41-29.03)	10.82 (4.31-27.14)	-	-	-
**Disability pension at some point**	-	-	-	4.78 (2.34-9.75)	4.77 (2.25-10.10)	4.68 (2.32-9.45)	4.71 (2.60-8.54)	4.29 (2.34-7.87)	4.67 (2.57-8.47)	-	-	-
**Disability pension at the end of the follow-up**	-	-	-	4.55 (2.06-10.04)	4.16 (1.82-9.50)	4.21 (1.95-9.09)	6.09 (3.09-11.99)	5.44 (2.69-10.98)	5.99 (3.03-11.84)	-	-	-

[1]Reference category,

[a]Model 1: adjusted for sex, [b]Model 2: adjusted for educational level, [c]Model 3: adjusted for any substance use disorder, [d]At 2019, [e]At June 2016, [f]At 2018

OR odds ratio, *CI* confidence interval
