# Supplementary material for: Effect of onset age on the long-term outcome of early-onset psychoses and other mental disorders: a register-based Northern Finland Birth Cohort 1986 study
Source: Eur Child Adolesc Psychiatry. 2023 Aug 11;33(6):1741–53. doi: 10.1007/s00787-023-02279-5 (PMC11211101; doi:10.1007/s00787-023-02279-5)
Supplement: Supplementary file 6 — Supplementary file6 (PDF 133 KB) [file 787_2023_2279_MOESM6_ESM.pdf]

European Child & Adolescent Psychiatry

Effect of onset age on the long-term outcome of early-onset psychoses and other mental disorders: a register based Northern Finland Birth Cohort 1986 study

Tuomas Majuri[1] · Marianne Haapea · Tanja Nordström · Veera Säynäjäkangas · Kristiina Moilanen · Jonna Tolonen · Leena Ala-Mursula · Jouko Miettunen · Erika Jääskeläinen

[1]Research Unit of Population Health, University of Oulu, Oulu, Finland.

Corresponding author:
M.D. Tuomas Majuri,
email tuomas.majuri@student.oulu.fi

Online supplement 6

Online supplement table 5. Characteristics of the sample in sensitivity analyses, those with psychosis or non-psychosis diagnosis before age 13 years excluded

Variable	**Psychosis 13–18 years (n=37)**	**Psychosis 18–22 years (n=61)**	**Non-psychotic psychiatric disorder 13–18 years (n=294)**	**Non-psychotic psychiatric disorder 18–22 years (n=377)**	**P13–18y vs. P18–22y, p-value**	**P13–18y vs. NP13–18y, p-value**	**P18–22y vs. NP18–22y, p-value**	**NP13–18y vs. NP18–22y, p-value**
**Sex, n (%)**					<0.001	0.032	0.014	0.002
Male	6 (16.2)	38 (62.3)	99 (33.7)	171 (45.4)				
Female	31 (83.8)	23 (37.7)	195 (66.3)	206 (54.6)				
**Hierarchical psychosis diagnosis, n (%)**[a]					0.150[b]			
Schizophrenia	7 (18.9)	8 (13.1)						
Schizophrenia spectrum disorder	4 (10.8)	1 (1.6)						
Affective psychosis	10 (27.0)	16 (26.2)						
Other non-affective psychosis	16 (43.2)	36 (59.0)						
**Non-psychotic psychiatric disorder diagnosis, n (%)**[a]								
Depression	16 (43.2)	24 (39.3)	118 (40.1)	171 (45.3)				
Bipolar disorder	4 (10.8)	4 (6.6)	1 (0.3)	11 (2.9)				
Anxiety disorder	10 (27.0)	20 (32.8)	75 (25.5)	188 (49.9)				
Alcohol use disorder	2 (5.4)	13 (21.3)	35 (11.9)	48 (12.7)				
Cannabis use disorder	0 (0.0)	1 (1.6)	2 (0.7)	6 (1.6)				
Other substance use disorder	1 (2.7)	7 (11.5)	7 (2.4)	22 (5.8)				
**Age of illness onset, psychosis, Md (IQR)**	16.1 (15.0-17.1)	20.6 (19.6-21.4)						
**Age of illness onset, non-psychotic psychiatric disorder, Md (IQR)**	15.3 (14.4-16.3)	19.8 (18.1-21.0)	15.8 (14.6-16.9)	20.3 (19.3-21.6)				
**Psychosis diagnosis at the end of the follow-up, n (%)**					0.393[b]	0.404[b]	0.229[b]	0.310[b]
Schizophrenia	9 (24.3)	19 (31.1)	4 (20.0)	6 (21.4)				

Schizophrenia spectrum disorder	5 (13.5)	3 (4.9)	4 (20.0)	4 (14.3)
Affective psychosis	10 (27.0)	13 (21.3)	2 (10.0)	9 (32.1)
Other non-affective psychosis	13 (35.1)	26 (42.6)	10 (50.0)	9 (32.1)

[a]During the time leading for belonging to the respective study group, [b]P-value presented by Fisher's exact test

Md median, *IQR* interquartile range
