# Supplementary material for: Effect of onset age on the long-term outcome of early-onset psychoses and other mental disorders: a register-based Northern Finland Birth Cohort 1986 study
Source: Eur Child Adolesc Psychiatry. 2023 Aug 11;33(6):1741–53. doi: 10.1007/s00787-023-02279-5 (PMC11211101; doi:10.1007/s00787-023-02279-5)
Supplement: Supplementary file 7 — Supplementary file7 (PDF 52 KB) [file 787_2023_2279_MOESM7_ESM.pdf]

European Child & Adolescent Psychiatry

Effect of onset age on the long-term outcome of early-onset psychoses and other mental disorders: a register based Northern Finland Birth Cohort 1986 study

Tuomas Majuri[1] · Marianne Haapea · Tanja Nordström · Veera Säynäjäkangas · Kristiina Moilanen · Jonna Tolonen · Leena Ala-Mursula · Jouko Miettunen · Erika Jääskeläinen

[1]Research Unit of Population Health, University of Oulu, Oulu, Finland.

Corresponding author:
M.D. Tuomas Majuri,
email tuomas.majuri@student.oulu.fi

Online supplement 7

Online supplement table 6. Psychosis conversion in the sensitivity analyses, those with psychosis or non-psychosis diagnosis before age 13 years excluded

	**Psychosis 13–18 years**				**Psychosis 18–22 years**			
	**Psychosis diagnosis at the end of the follow-up**				**Psychosis diagnosis at the end of the follow-up**			
	Schizophrenia	Schizophrenia spectrum disorder	Affective psychosis	Other non-affective psychosis	Schizophrenia	Schizophrenia spectrum disorder	Affective psychosis	Other non-affective psychosis
**Hierarchical psychosis diagnosis**								
Schizophrenia	7	0	0	0	8	0	0	0
Schizophrenia spectrum disorder	1	3	0	0	0	1	0	0
Affective psychosis	0	1	9	0	4	1	11	0
Other non-affective psychosis	1	1	1	13	7	1	2	26
