# Supplementary material for: Effect of onset age on the long-term outcome of early-onset psychoses and other mental disorders: a register-based Northern Finland Birth Cohort 1986 study
Source: Eur Child Adolesc Psychiatry. 2023 Aug 11;33(6):1741–53. doi: 10.1007/s00787-023-02279-5 (PMC11211101; doi:10.1007/s00787-023-02279-5)
Supplement: Supplementary file 8 — Supplementary file8 (PDF 65 KB) [file 787_2023_2279_MOESM8_ESM.pdf]

European Child & Adolescent Psychiatry

Effect of onset age on the long-term outcome of early-onset psychoses and other mental disorders: a register based Northern Finland Birth Cohort 1986 study

Tuomas Majuri[1] · Marianne Haapea · Tanja Nordström · Veera Säynäjäkangas · Kristiina Moilanen · Jonna Tolonen · Leena Ala-Mursula · Jouko Miettunen · Erika Jääskeläinen

[1]Research Unit of Population Health, University of Oulu, Oulu, Finland.

Corresponding author:
M.D. Tuomas Majuri,
email tuomas.majuri@student.oulu.fi

Online supplement 8

Online supplement table 7. Clinical outcomes during the follow-up in the sensitivity analyses, those with psychosis or non-psychosis diagnosis before age 13 years excluded, frequencies, percentages, p-values and unadjusted odds ratios in relation to the reference groups (=1)

Variable	**Psychosis 13–18 years (n=37)**	**Psychosis 18–22 years (n=61)**	**Non-psychotic psychiatric disorder 13–18 years (n=294)**	**Non-psychotic psychiatric disorder 18–22 years (n=377)**	**P13-18y vs. P18–22y[1]**		**P13-18y vs. NP13-18y[1]**		**P18–22y vs. NP18–22y[1]**		**NP13-18y vs. NP18–22y[1]**	
					**Crude OR (95% CI)**	**p-value**	**Crude OR (95% CI)**	**p-value**	**Crude OR (95% CI)**	**p-value**	**Crude OR (95% CI)**	**p-value**
**Psychiatric hospital episodes, psychosis, n (%)[a]**	4 (10.8)	14 (23.0)	10 (3.4)	12 (3.2)	0.41 (0.12-1.35)	0.141	3.44 (1.02-11.59)	0.046	9.06 (3.96-20.75)	<0.001	1.07 (0.46-2.51)	0.875
**Psychiatric hospital episodes, any psychiatric, n (%)[a]**	6 (16.2)	20 (32.8)	28 (9.5)	46 (12.2)	0.40 (0.14-1.11)	0.077	1.84 (0.71-4.79)	0.212	3.51 (1.89-6.51)	<0.001	0.76 (0.46-1.25)	0.273
**Substance use disorders during the follow-up, n (%)**												
Any substance use disorder, n (%)	6 (16.2)	23 (37.7)	75 (25.5)	89 (23.6)	0.32 (0.12-0.88)	0.028	0.57 (0.23-1.41)	0.220	1.96 (1.11-3.46)	0.021	1.11 (0.78-1.58)	0.569
Alcohol use disorder, n (%)	4 (10.8)	19 (31.1)	60 (20.4)	65 (17.2)	0.27 (0.08-0.86)	0.027	0.47 (0.16-1.39)	0.172	2.17 (1.19-3.97)	0.012	1.23 (0.83-1.82)	0.296
Cannabis use disorder, n (%)	1 (2.7)	3 (4.9)	6 (2.0)	12 (3.2)	0.54 (0.05-5.36)	0.596	1.33 (0.16-11.39)	0.793	1.57 (0.43-5.75)	0.493	0.63 (0.24-1.71)	0.367
Other substance use disorder, n (%)	4 (10.8)	10 (16.4)	32 (10.9)	46 (12.2)	0.62 (0.18-2.14)	0.447	0.99 (0.33-2.98)	0.989	1.41 (0.67-2.97)	0.365	0.88 (0.54-1.42)	0.598

[1]Reference category

[a]Psychiatric hospital episodes counted only for the last five years of follow-up (2015-2019)

OR odds ratio, *CI* confidence interval
