# Supplementary material for: Effect of onset age on the long-term outcome of early-onset psychoses and other mental disorders: a register-based Northern Finland Birth Cohort 1986 study
Source: Eur Child Adolesc Psychiatry. 2023 Aug 11;33(6):1741–53. doi: 10.1007/s00787-023-02279-5 (PMC11211101; doi:10.1007/s00787-023-02279-5)
Supplement: Supplementary file 9 — Supplementary file9 (PDF 77 KB) [file 787_2023_2279_MOESM9_ESM.pdf]

European Child & Adolescent Psychiatry

Effect of onset age on the long-term outcome of early-onset psychoses and other mental disorders: a register based Northern Finland Birth Cohort 1986 study

Tuomas Majuri[1] · Marianne Haapea · Tanja Nordström · Veera Säynäjäkangas · Kristiina Moilanen · Jonna Tolonen · Leena Ala-Mursula · Jouko Miettunen · Erika Jääskeläinen

[1]Research Unit of Population Health, University of Oulu, Oulu, Finland.

Corresponding author:
M.D. Tuomas Majuri,
email tuomas.majuri@student.oulu.fi

Online supplement 9

Online supplement table 8. Work-family outcomes during the follow-up in the sensitivity analyses, those with psychosis or non-psychosis diagnosis before age 13 years excluded, frequencies, percentages, p-values and unadjusted odds ratios in relation to the reference groups (=1)

Variable	**Psychosis 13–18 years (n=37)**	**Psychosis 18–22 years (n=61)**	**Non-psychotic psychiatric disorder 13–18 years (n=294)**	**Non-psychotic psychiatric disorder 18–22 years (n=377)**	**P13-18y vs. P18–22y[1]**		**P13-18y vs. NP13-18y[1]**		**P18–22y vs. NP18–22y[1]**		**NP13-18y vs. NP18–22y[1]**	
					**Crude OR (95% CI)**	**p-value**	**Crude OR (95% CI)**	**p-value**	**Crude OR (95% CI)**	**p-value**	**Crude OR (95% CI)**	**p-value**
**Educational level, n (%)[a]**												
Basic or below	8 (21.6)	18 (29.5)	59 (19.2)	60 (15.9)	1		1		1		1	
Secondary	20 (54.1)	35 (57.4)	164 (55.8)	213 (56.5)	1.29 (0.47-3.49)	0.622	0.90 (0.38-2.15)	0.812	0.55 (0.29-1.04)	0.064	0.78 (0.52-1.18)	0.246
Tertiary	9 (24.3)	8 (13.1)	71 (24.1)	104 (27.6)	2.53 (0.71-8.97)	0.150	0.94 (0.34-2.58)	0.896	0.26 (0.11-0.63)	0.003	0.69 (0.43-1.11)	0.127
**Marital status, n (%)[b]**						0.001				0.001		0.992
Single/divorced/separated/widowed	24 (64.9)	58 (95.1)	213 (72.4)	273 (72.4)	1		1		1		1	
Married/registered	13 (35.1)	3 (4.9)	81 (27.6)	104 (27.6)	10.47 (2.74-40.09)		1.42 (0.69-2.93)	0.337	0.14 (0.04-0.44)		1.00 (0.71-1.40)	
**Having children, n (%)[b]**	16 (43.2)	13 (21.3)	145 (49.3)	181 (48.0)	2.81 (1.15-6.88)	0.023	0.78 (0.39-1.56)	0.487	0.29 (0.15-0.56)	<0.001	1.05 (0.78-1.43)	0.736
**Socio-economic status, n (%)[c]**												
White collar	12 (32.4)	10 (16.9)	107 (37.7)	138 (38.3)	1		1		1		1	
Farmer/entrepreneur/manual worker/student/other	14 (37.8)	30 (50.8)	156 (54.9)	201 (55.8)	0.39 (0.14-1.11)	0.078	0.80 (0.36-1.80)	0.589	2.06 (0.98-4.35)	0.058	1.00 (0.72-1.39)	0.995
Pensioner	11 (29.7)	19 (32.2)	21 (7.4)	21 (5.8)	0.48 (0.16-1.48)	0.202	4.67 (1.82-11.99)	0.001	12.49 (5.11-30.49)	<0.001	1.29 (0.67-2.48)	0.447

**Disability pension at some point, n (%)**	15 (40.5)	27 (45.8)	35 (12.3)	53 (14.4)	0.81 (0.35-1.86)	0.616	4.85 (2.30-10.22)	<0.001	5.00 (2.77-9.01)	<0.001	0.83 (0.53-1.32)	0.434
**Disability pension at the end of the follow-up, n (%)**	11 (29.7)	20 (33.9)	25 (8.8)	26 (7.2)	0.83 (0.34-2.00)	0.671	4.38 (1.40-9.91)	<0.001	6.59 (3.37-12.88)	<0.001	1.24 (0.70-2.20)	0.461

[1]Reference category

[a]At 2019, [b]At June 2016, [c]At 2018

OR odds ratio, *CI* confidence interval
